# Altered ATP release and metabolism in dorsal root ganglia of neuropathic rats

**DOI:** 10.1186/1744-8069-4-66

**Published:** 2008-12-24

**Authors:** Yoshizo Matsuka, Takeshi Ono, Hirotate Iwase, Somsak Mitrirattanakul, Kevin S Omoto, Ting Cho, Yan Yan N Lam, Bradley Snyder, Igor Spigelman

**Affiliations:** 1 Division of Oral Biology & Medicine, School of Dentistry, University of California, Los Angeles, California, USA; 2 Department of Oral and Maxillofacial Rehabilitation, Graduate School of Medicine, Dentistry and Pharmaceutical Sciences, Okayama University, Okayama, Japan; 3 Department of Education, Japan Ground Self-Defense Force Medical School, Tokyo, Japan; 4 Faculty of Dentistry, Mahidol University, Bangkok, Thailand

## Abstract

**Background:**

Adenosine 5'-triphosphate (ATP) has a ubiquitous role in metabolism and a major role in pain responses after tissue injury. We investigated the changes in basal and KCl-evoked ATP release from rat dorsal root ganglia (DRG) after peripheral neuropathy induction by unilateral sciatic nerve entrapment (SNE).

**Results:**

After SNE, rats develop long-lasting decreases in ipsilateral hindpaw withdrawal thresholds to mechanical and thermal stimulation. At 15–21 days after neuropathy induction, excised ipsilateral L4-L5 DRG display significantly elevated basal extracellular ATP levels compared to contralateral or control (naive) DRG. However, KCl-evoked ATP release is no longer observed in ipsilateral DRG. We hypothesized that the differential SNE effects on basal and evoked ATP release could result from the conversion of extracellular ATP to adenosine with subsequent activation of adenosine A1 receptors (A1Rs) on DRG neurons. Adding the selective A1R agonist, 2-chloro-N^6^-cyclopentyladenosine (100 nM) significantly decreased basal and evoked ATP release in DRG from naïve rats, indicating functional A1R activation. In DRG ipsilateral to SNE, adding a selective A1R antagonist, 8-cyclopentyl-1,3-dipropylxanthine (30 nM), further increased basal ATP levels and relieved the blockade of KCl-evoked ATP release suggesting that increased A1R activation attenuates evoked ATP release in neurons ipsilateral to SNE. To determine if altered ATP release was a consequence of altered DRG metabolism we compared O_2 _consumption between control and neuropathic DRG. DRG ipsilateral to SNE consumed O_2 _at a higher rate than control or contralateral DRG.

**Conclusion:**

These data suggest that peripheral nerve entrapment increases DRG metabolism and ATP release, which in turn is modulated by increased A1R activation.

## Background

Certain neuropathic pain states that result from peripheral nerve injury are associated with hyperexcitability of neurons within sensory ganglia [1] and potentiated cross-excitation among neighboring sensory neurons in the absence of synaptic specializations [2]. Cross-excitation occurs when discharge in one sensory neuron leads to a depolarization in the cell bodies of adjacent passive neurons sharing the same ganglion. Cross-excitation was reported to be chemically mediated [3], but the identity of the chemical mediator(s) is unknown. One possible candidate is ATP which, in addition to its universal role in metabolism, is released from stimulated sensory nerves [4]. There is compelling evidence for exocytotic vesicular release of ATP from neurons [5] and we have demonstrated Ca^2+^-dependent ATP release within trigeminal ganglia after neuronal stimulation *in vivo*, as well as from isolated somata of trigeminal sensory neurons [6]. For ATP release from non-neuronal cells, a variety of transport mechanisms have been proposed, including but not limited to ATP-binding cassette transporters and connexin hemichannels, as well as vesicular release [5]. Extracellular ATP levels are controlled by both specific (ectonucleotidases) and nonspecific (ectoenzymes) enzymes with an extracellularly-oriented catalytic site [7]. Other ectoenzymes also contribute to interconversion of nucleotides, exerting control over the extracellular levels of various nucleotides under physiological and pathophysiological conditions [7]. Most studies examined ATP release after bulk equilibration in extracellular samples with the luciferin-luciferase assay (e.g., [6]). While highly sensitive, these measurements greatly underestimate the true [ATP] in the pericellular environment because of its rapid degradation by ectonucleotidases and delayed diffusion due to unstirred layer effects [8]. Recent studies have suggested that true levels of ATP at the plasma membrane are underestimated by >20-fold with bulk phase measurements [9]. This should be considered when relating the extracellularly measured concentrations of nucleotides to the effects on their receptors.

ATP and related purines interact with three families of purinergic receptors, the ionotropic P2X (7 receptors cloned) and metabotropic P2Y (8 receptors cloned) and P1 (4 receptors cloned) [8,10-12]. Given such tremendous diversity of receptors and natural ligands it is not surprising that ATP and related purines might be involved in a wide spectrum of physiological and pathophysiological activities, including nociception and chronic pain. For example, ATP is a known algogen whose application by iontophoresis to human skin elicits pain which is exaggerated by inflammation [13]. Application of ATP to dissociated neurons from rat sensory ganglia evokes inward currents via the P2X ion channels, mainly P2X2/3 purinoceptors (P2XRs) [14,15]. Sensory neurons in culture form synapses that appear to utilize ATP as their neurotransmitter [16] and injury of skin cells near cultured neurons results in P2XR activation on identified nociceptors [17]. The P2X3Rs are especially concentrated in small diameter sensory neurons and their expression is differentially affected in injured and uninjured neurons after peripheral neuropathy induction [18-20]. Increased P2XR-mediated signaling appears to contribute to sensory neuron hyperexcitability after spinal nerve ligation [21,22], while functional down-regulation of P2X3Rs alleviates neuropathy symptoms [23]. The role of P2YRs in nociception and chronic pain is less clear. Several studies have suggested that ATP or uridine 5'-triphosphate (UTP) acting via P2Y2Rs increase the excitability and action potential discharge of sensory neurons, promote neuropeptide release and axonal transport, and facilitate increased expression of growth factors and neuropeptides by increasing the phosphorylation of cAMP responsive element binding protein [24]. However, there is also evidence that activation of P2Y1Rs on sensory neurons inhibits N-type voltage-gated Ca^2+ ^channels (VGCCs) [25] and P2X3R channels [26], while intrathecal administration of preferential agonists for P2Y1 and P2Y2/4 receptor subtypes inhibits pain transmission [8,26]. Aside from the reported increase in P2Y1R mRNA after sciatic nerve transection [27], little is known about the plasticity of P2YR signaling in neuropathic pain states.

Involvement of the P1 receptors in nociception and chronic pain is well-established. A1 and A3R activation usually results in antinociception and decreased neuropathic pain symptoms, whereas A2A and A2BR activation promotes nociception [10,28]. These receptors respond selectively to extracellular adenosine produced from two sources: 1) bidirectional facilitated diffusion and 2) extracellular conversion of adenine nucleotides [10,29]. The bidirectional transport of adenosine into and out of cells is dependent on the intra and extracellular levels of adenosine [30]. Blockade by transport inhibitors usually increases extracellular adenosine levels. Inhibition of cytosolic adenosine kinase also increases extracellular adenosine [10,29]. Spinal nerve ligation enhances stimulus-evoked increases in extracellular adenosine [31]. Inhibitors of adenosine kinase appear to decrease pain symptoms of peripheral neuropathy in preclinical studies [32].

By contrast, neuropathy-induced changes in extracellular levels of ATP (an adenosine precursor), have not been addressed. We used extracellular sampling from an *in vitro *dorsal root ganglion (DRG) preparation [33] to assess possible changes in ATP release from DRG in a rat model of neuropathy induced by sciatic nerve entrapment [34] and examine possible mechanisms and consequences of such alterations. We found that basal extracellular [ATP] was increased while KCl-evoked ATP release was suppressed in the injured DRG. Selective blockade of A1Rs increased basal [ATP] and relieved the block of KCl-evoked ATP release. We also demonstrated that cellular metabolism was increased in the injured DRG, representing a potential source of neuropathy-induced increases in extracellular [ATP].

## Materials and methods

All experimental procedures were carried out in accordance with the guidelines of the NIH on animal care and University of California at Los Angeles animal research committee.

### Sciatic nerve entrapment (SNE)

Surgery was performed as described previously [34]. Adult male Sprague-Dawley rats weighing 200–250 g were used. Rats were anesthetized (pentobarbital 50 mg/kg, i.p.) or alternatively with a mixture of isoflurane (1–3%), N_2_O (50%), and O_2 _(inhalation) and the subsequent surgical procedures were performed under sterile conditions. The hair of the lower back and thigh of the rats was shaved and the skin was sterilized with povidone-iodine solution. A skin incision was made on one thigh and the sciatic nerve exposed through blunt dissection of the overlying muscle. Three Tygon^® ^cuffs (length = 1 mm, outer diameter = 2.28 mm, inner diameter = 0.76 mm) were placed around the exposed sciatic nerve. This tubing size allowed them to be fitted snugly around the sciatic nerve without constricting it. The muscle layer was closed with absorbable sutures (5.0 Vicryl, Ethicon, Johnson & Johnson, Irvine, CA) and the skin was closed with suture (3.0 Nylon, Ethicon). Triple antibiotic cream (Bacitracin, Neomycin and Polymyxin B, Rite Aid, Harrisburg, PA) was applied over the wound site. Buprenorphine (0.01 mg/kg, s.c.) was injected once daily for 2 days for post-surgical analgesia. Sutures were removed 7–10 days post-surgery under isoflurane/N_2_O anesthesia.

### Behavioral testing

Mechanical sensitivity was assessed using an electronic von Frey hair pressure transducer (IITC Instruments, Model 1601C, Woodland Hills, CA). Baseline behavioral testing was performed one day before and on the same day prior to surgery. Testing was repeated on post-operative days 1, 2, 3, 4, 5, 7, 9, 11, 13 and 15. The rat was gently placed in a plastic-walled cage (10 × 20 × 13 cm) with a metal mesh floor (0.6 × 0.6 cm holes). A point in the middle of the hindpaw was tested with the tip of the electronic von Frey hair pressed on the spot until the animal withdrew the foot. The force (g) applied at the time of withdrawal was recorded. Each hindpaw was tested three times at 1 min intervals and the results were averaged for each paw for that day.

Thermal sensitivity testing was performed using the Hargreaves paw withdrawal apparatus (Hargreaves Model 390, IITC Instruments) which measures the withdrawal latency from a radiant heat source directed at the proximal half of the plantar surface of each hindpaw. The baseline and post-operative testing schedule was the same as for mechanical sensitivity. Prior to testing, rats were allowed to acclimate to the testing environment for 15 min, which consisted of translucent plastic-walled individual chambers (10 × 20 × 20 cm) and a 3 mm thick glass bottom (preheated to 30°C). A radiant heat source consisting of an adjustable infrared lamp and a built-in stopwatch accurate to 0.1 s were used to measure paw withdrawal latency. Each paw was tested three times at 25% maximal heat intensity allowing 5 min between each test. The test was performed only when a rat was stationary and standing on all four paws. Special care was taken to keep the glass bottom clean and dry during the testing. If the glass needed to be cleaned during the experiment, the rats were allowed 5–10 min to reacclimatize to the environment. The results of three tests were averaged for each paw for that day.

### DRG extraction and sample collection

At 15–21 days after SNE rats were anesthetized with sodium pentobarbital (50 mg/kg, i.p.) or alternatively with a mixture of isoflurane (1–3%), N_2_O (50%), and O_2 _(inhalation). The L4 and L5 DRG on both sides were excised with attached spinal nerves and dorsal roots, and placed in cold (0–4°C) low-Na^+ ^artificial cerebrospinal fluid (ACSF) composed of (in mM): KCl 2.5, NaH_2_PO_4 _1.25, CaCl_2 _0.5, MgCl_2 _3.5, NaHCO_3 _26, glucose 10 and sucrose 248. The ACSF was continuously bubbled with a 95/5% mixture of O_2_/CO_2 _to ensure adequate oxygenation of ganglia and pH 7.4. The DRG were cleaned of surrounding connective tissue and incubated for 5 minutes at room temperature in low-Na^+ ^artificial cerebrospinal fluid (ACSF) composed of (in mM): NaCl 63, KCl 2.5, NaH_2_PO_4 _1.25, CaCl_2 _0.5, MgCl_2 _3.5, NaHCO_3 _26, glucose 10 and sucrose 124 before being placed in the sample collection chamber where they were slowly (160 μl/min) perfused with normal ACSF (NaCl 125 and no sucrose) at 35.0 ± 0.5°C. In initial experiments, we used an open sample collection chamber (custom-made), where DRG could be monitored for physiological integrity using extracellular suction electrode recordings of evoked compound action potentials [33]. In subsequent experiments with adenosine receptor ligands, we switched to a commercial closed chamber (RC-30WA, Warner Instruments) for better flow and temperature control. High KCl-containing (100 mM) ACSF was applied after the baseline collection. For high K^+ ^solutions, NaCl content was reduced to maintain ACSF osmolality. 8-Cyclopentyl-1,3-dipropylxanthine (DPCPX, 10 μM in 10% dimethylsulfoxide) and N^6^-cyclopentyladenosine (NCPA,10 μM in H_2_O) from frozen (-20°C) stock were diluted in ACSF just prior to perfusion. In these experiments, the comparison perfusate without DPCPX contained 0.03% dimethylsulfoxide.

Samples were collected at 1 min intervals into polypropylene tubes (0.2 ml; Corning Incorporated, Corning, NY), immediately frozen in liquid nitrogen and stored at -80°C until analysis. All experimental samples were analyzed within 3 days of collection.

### Measurement of ATP release

Release of ATP was determined directly using the firefly luciferin-luciferase (L/L) assay. In initial experiments, L/L reagent (30 μL, CLS II, Boehringer Mannheim, Mannheim, Germany) was added to samples (40 μL) in a cylindrical cuvette, mixed and placed into a luminometer (TD-20/20, Turner Designs, Sunnyvale, CA). Chemiluminometer settings were delay: 1 sec, integration: 10 sec. In experiments with adenosine receptor ligands, samples were placed in closed-bottom 96-well white polystyrene plates (Corning Life Sciences, Lowell, MA) in a microplate reader (Synergy HT, Biotek, Winooski, VT) which automatically dispensed and mixed L/L solutions (ATP Bioluminescence kit HS II, Roche Diagnostics, Indianapolis, IN) to individual wells. Several concentrations of ATP standard (100 pM – 1 μM) with ACSF were measured before analysis of each experimental sample set. Separate ATP standards containing appropriate KCl and/or drug concentrations were also measured when appropriate. Microplate reader settings were delay: 0 sec, integration: 15 sec.

### Measurement of O_2 _consumption

The L4 and L5 DRG were obtained from anesthetized rats as described above and the attached nerves were cut at 10 mm from each side of the DRG. After 15 min incubation in cold normal ACSF, both DRG were transferred into a closed chamber (Multi-Port Measurement Chamber; World Precision Instrument, Sarasota, FL) filled with 2.5 ml of N-2-hydroxyethylpiperazine-N'-2-ethanesulphonic acid (HEPES, 26 mM)-buffered ACSF. Oxygen consumption was measured with an oxygen probe (ISO2; World Precision Instruments, Sarasota, FL) which continuously measured dissolved oxygen in HEPES-buffed ACSF. Once the chamber temperature reached 34.5 ± 0.4°C, recording continued for 60 min and later analyzed off-line using the pCLAMP9 software (Molecular Devices, Union City, CA).

### Statistical analysis

All data are presented as mean ± S.E.M. Statistical analyses (one-way repeated measures ANOVA with post hoc comparisons, one-way ANOVA and two-way ANOVA) were used to compare the data.

## Results

### Hindpaw withdrawal thresholds after sciatic nerve entrapment (SNE)

**Figure 1:**
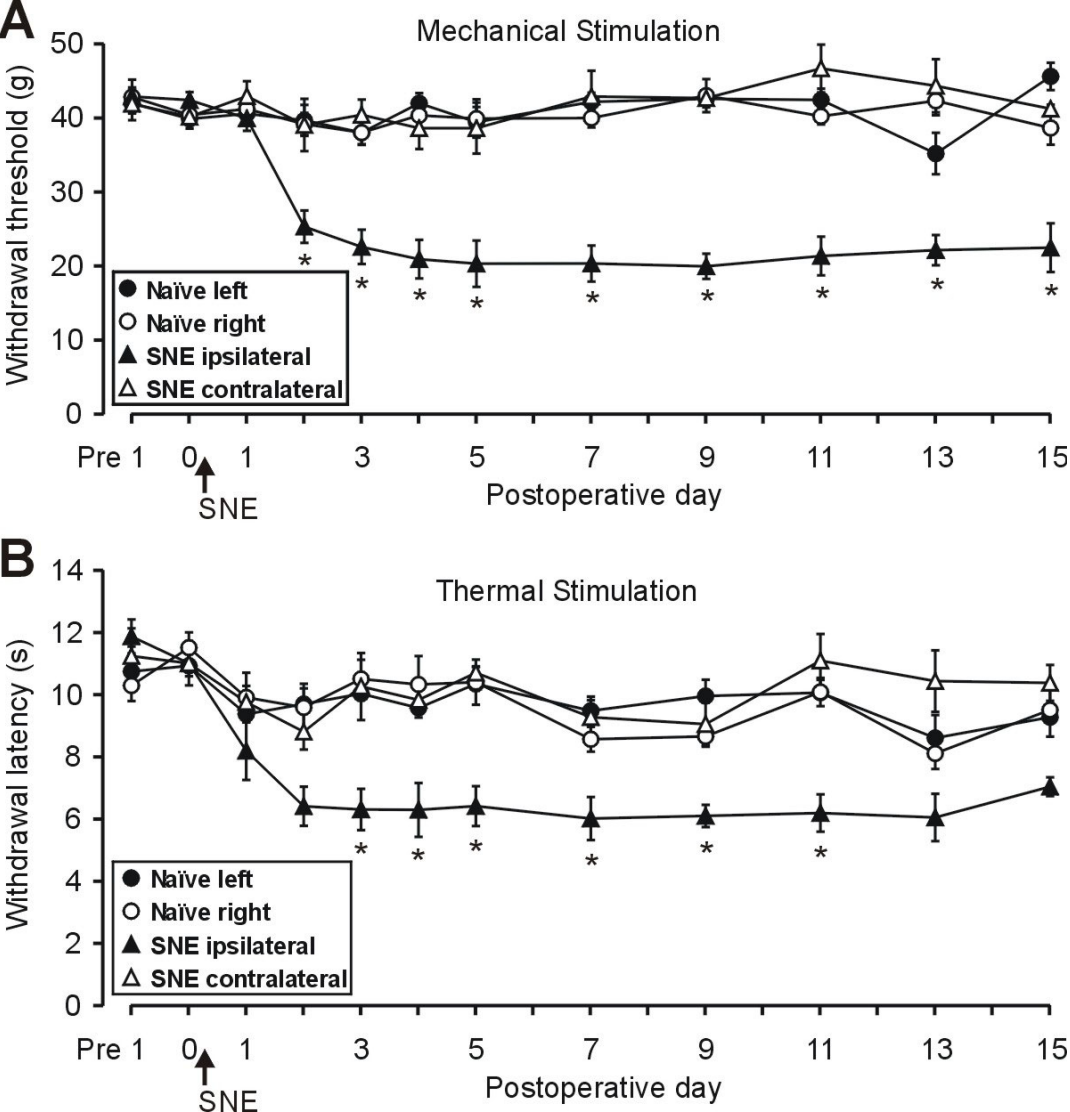
**Increased mechanical and thermal sensitivity after SNE induction**. A: hindpaw withdrawal thresholds (mean ± S.E.M.) to mechanical stimuli on the ipsilateral side (n = 6 rats) are significantly decreased compared to contralateral side and naïve rats (n = 6) at 2 days after SNE surgery. Note that the decreases persist to the last day of measurements. B: hindpaw withdrawal latency (mean ± S.E.M.) from thermal stimuli on the ipsilateral side is also significantly reduced by the 3^rd ^day after SNE. *, significant difference from other group means.

SNE is a variation on chronic constriction injury in which the loose ligatures placed around the sciatic nerve are replaced by fixed-diameter polyethylene cuffs, resulting in decreased variability across animals [34]. The use of chemically inert polyethylene also prevents potentially confounding inflammatory responses that can occur in response to chromic catgut sutures. Stable baseline measurements were obtained 1 day pre-operatively and on the day just prior to the operation to both mechanical and thermal stimuli. Post-operatively (PO), measurements revealed that the SNE animals developed increased sensitivity of ipsilateral hindpaw to both types of stimuli (Fig. 1). For mechanical sensitivity, the baseline threshold for withdrawal was 40–45 g prior to surgery, and this was maintained PO for the contralateral paw and in naïve (untreated) rats (Fig. 1A). The threshold for the ipsilateral side dropped significantly to ~20 g by 2 days PO; a change that was maintained throughout the testing period. Significant decreases in latency to hindpaw withdrawal from a thermal stimulus developed by 3 days PO, and the change in latency was maintained throughout the testing period (Fig. 1B). There were also signs of spontaneous pain behaviors which included guarding behavior and changes in the posture of the affected hindpaw such as plantar flexion and toe-clenching, typical of this model [34].

### Basal ATP release from L4/L5 DRG after SNE

**Figure 2:**
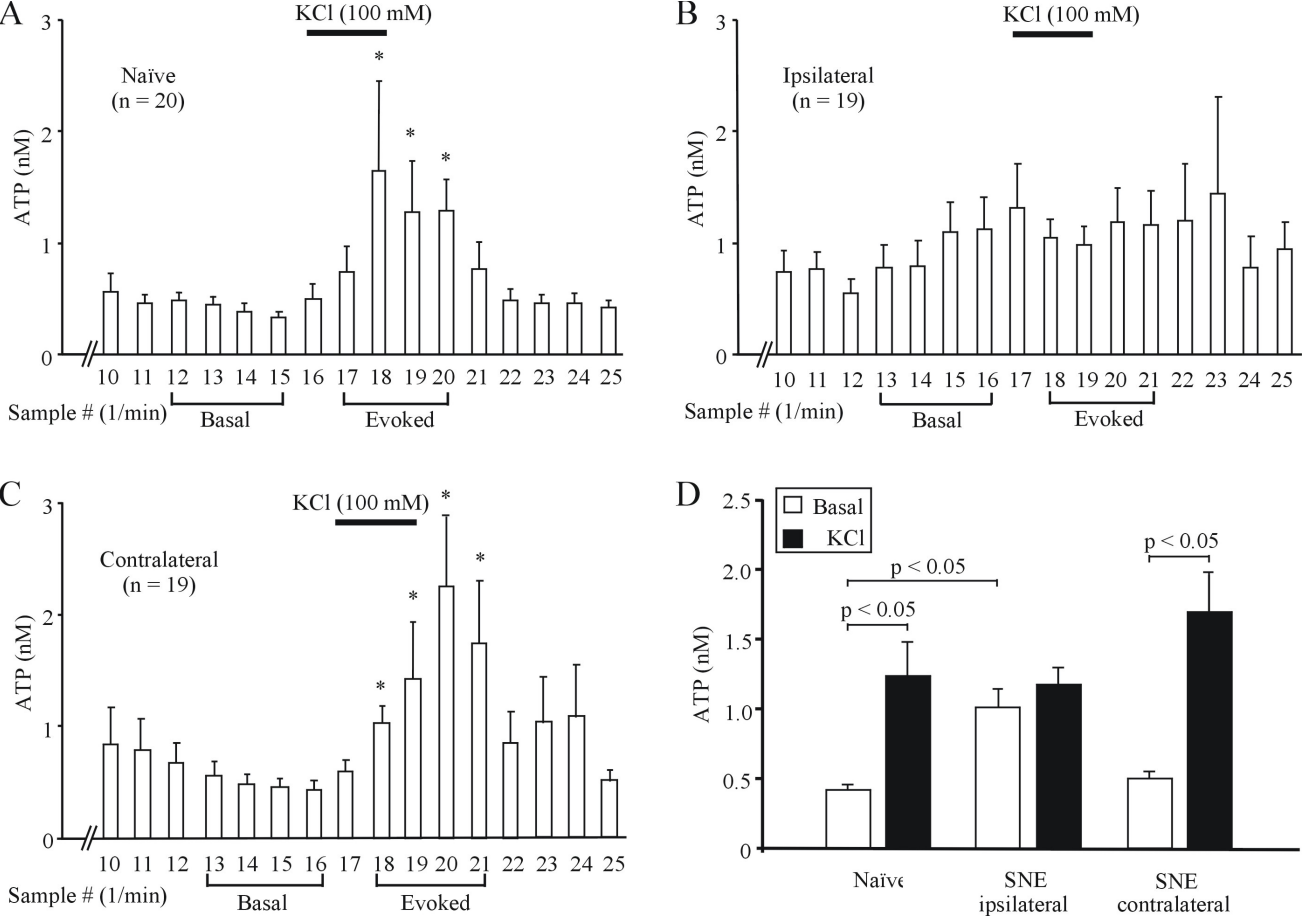
**Altered ATP release from DRG after SNE injury**. A: ATP release from naive rat DRG is increased by KCl (100 mM) stimulation. * denotes p < 0.05 compared with basal ATP levels. Basal release was defined as the average of 4 samples obtained 1 min prior to onset of KCl stimulation. For clarity, samples 1–9 are not shown. B: Basal ATP release is higher than in naive DRG, whereas KCl stimulation no longer evokes ATP release from ipsilateral DRG. C: The pattern of ATP release from contralateral DRG is similar to naive DRG. * denotes p < 0.05 compared with basal ATP level. D: Summary graph of basal and evoked ATP release in DRG from naive and SNE neuropathic rats. Data were binned as illustrated in A-C. Note the increased basal and loss of KCl-evoked ATP release in ganglia ipsilateral to the SNE.

Sciatic nerve entrapment leads to axonal injury of neurons with cell bodies in both L4 and L5 DRG [34], prompting us to measure ATP release in both L4 and L5 DRG. In these initial studies, samples collected for the first few minutes of DRG perfusion had very high ATP content (not shown) and declined to stable levels by 12–15 min of perfusion. These initial high levels of ATP are probably due to mechanical deformation of the DRG preparation [35] when it was placed in the collection chamber and therefore are not included in the experimental analysis. In some of these experiments we also confirmed the physiological integrity of the DRG preparations by measuring evoked compound action potentials. The fitting of suction recording electrodes to spinal nerves and dorsal roots likely also contributed to the initial high ATP levels. After establishing a stable baseline, perfusion of naive rat L4 and L5 DRG (n = 20, 10 rats) with 100 mM KCl evoked a reversible increase in the amount of ATP (Fig. 2A). In a separate set of experiments, we could not detect KCl-evoked increases in ATP release from preparations containing either spinal nerves alone or dorsal roots alone (data not shown), indicating that DRG neuronal somata are required for KCl-evoked ATP release. Also, we demonstrated previously that KCl-evoked ATP release *in vivo *is dependent on calcium influx through voltage-gated calcium channels because it could be blocked by cadmium application [6]. Comparison of the *in vitro *experimental data indicated that baseline samples from ipsilateral DRG of SNE rats (n = 19, 11 rats) had significantly (*p *< 0.05) higher ATP levels than samples from control DRG. However, 100 mM KCl perfusion did not evoke ATP release in ipsilateral DRG as it did in naive rat DRG (Fig. 2B, D). By contrast, samples from contralateral DRG had basal levels of ATP similar to DRG from naive rats and exhibited robust increases in ATP content after 100 mM KCl perfusion (Fig. 2C, D).

### Effect of selective A1R activation on basal and KCl-evoked ATP release

**Figure 3:**
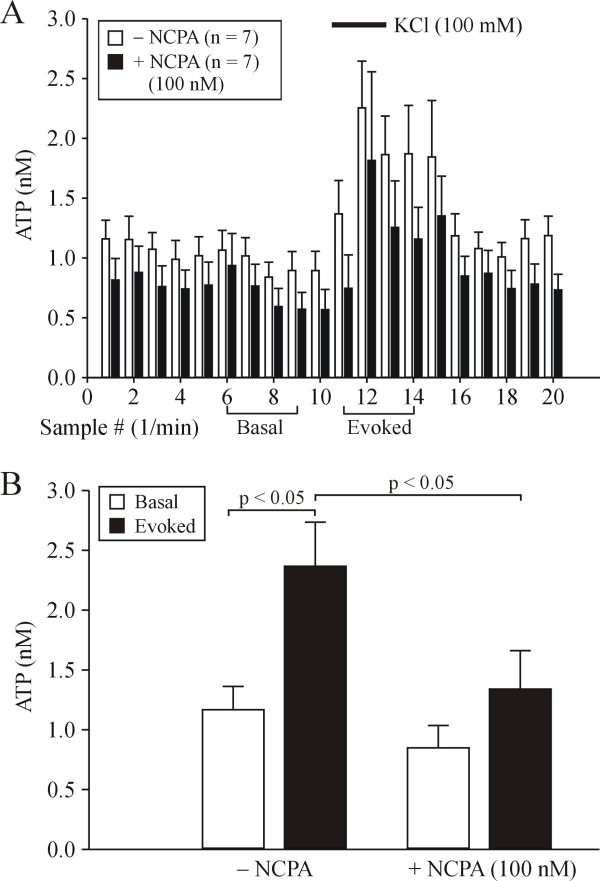
**Selective A1 receptor activation decreases basal and KCl-evoked ATP release**. A: Data are presented as mean ± SEM of sample ATP levels from naïve rat DRG in normal perfusate (white bars) and in the continuous presence of NCPA (100 nM, black bars). When DRG are perfused in the presence of NCPA (100 nM) both basal and evoked ATP release is visibly decreased. * denotes p < 0.05 compared with basal ATP levels. B: Summary graph of basal and evoked ATP release in DRG from naive rats with and without NCPA. Data were binned as illustrated in A.

The differential effects of SNE on basal and evoked ATP release suggested a possible involvement of ATP metabolites. Normally, extracellular ATP is regulated by the activity of various ectonucleotidases which convert ATP to di- and mono-phosphates and subsequently into adenosine. DRG neurons are endowed with A1Rs whose activation, in turn, leads to decreased voltage-gated Ca^2+ ^channel (VGCC) activity [36]. Since KCl-evoked ATP release in sensory neurons is dependent on VGCC activation [6], we hypothesized that the block of evoked ATP release after SNE could result from the conversion of extracellular ATP to adenosine with subsequent activation of A1Rs on DRG neurons. To test this hypothesis we first had to demonstrate that A1R activation can lead to altered ATP release within DRG. To that end, we compared basal and KCl-evoked ATP release in DRG from naïve rats in the presence and absence of the selective A1R agonist, NCPA (100 nM). In the absence of NCPA (n = 6 DRG, 4 rats), KCl (100 mM) application reliably evoked a reversible increase in the sample ATP levels (Fig. 3A, B). However, in the continuous presence of NCPA (n = 7 DRG, 4 rats), there was a significant decrease in both basal and KCl-evoked ATP release (Fig. 3A, B), suggesting functional modulation of ATP release by A1R activation.

### Effect of selective A1R blockade on basal and KCl-evoked ATP release in DRG ipsilateral to SNE

**Figure 4:**
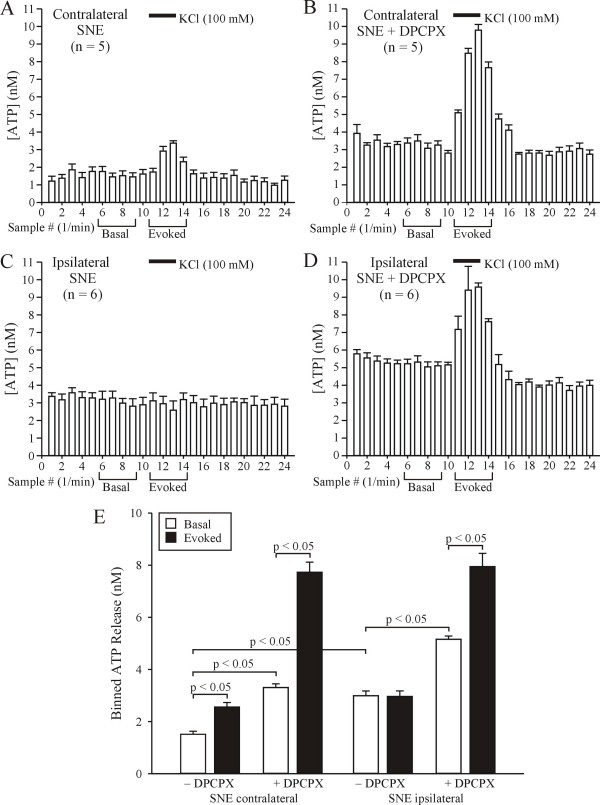
**Selective A1 receptor blockade increases basal ATP levels and relieves the blockade of KCl-evoked ATP release in DRG ipsilateral to SNE**. A: Data are presented as mean ± SEM of sample ATP concentration from L4/L5 DRG contralateral to SNE before and after KCl (100 mM) application. B: Data from DRG contralateral to SNE during continuous presence of DPCPX (30 nM). C: Data from DRG ipsilateral to SNE before and after KCl (100 mM) application. Note the absence of KCl-evoked ATP release. D: Data from DRG ipsilateral to SNE during continuous presence of DPCPX (30 nM). E: Summary graph of basal and evoked ATP release in DRG from neuropathic rats in the presence and absence of DPCPX. Data were binned as illustrated in A-D. Note the DPCPX-induced increases in basal release and recovery of KCl-evoked release of ATP in DRG ipsilateral to SNE.

We next tested our hypothesis by measuring basal and KCl-evoked ATP release in DRG from neuropathic rats in the presence and absence of the selective A1R antagonist, DPCPX. DRG ipsilateral to SNE exhibited higher basal ATP concentrations than contralateral DRG (compare Fig. 4A with 4C). Also, there was no significant KCl-evoked ATP release in ipsilateral DRG compared with the contralateral DRG, confirming our initial findings (see Fig. 2). As we suspected, in the presence of the selective A1R inhibitor, DPCPX (30 nM), both basal and KCl-evoked ATP release was significantly increased in DRG contralateral to SNE (compare Fig. 4A with 4B). Blockade of A1Rs in DRG ipsilateral to SNE further increased basal ATP levels compared to untreated ipsilateral SNE DRG (compare Fig. 4C with 4D). In support of our hypothesis, we observed a robust KCl-evoked release of ATP in DRG ipsilateral to SNE during A1R blockade (Fig. 4D and 4E).

### Altered metabolism after SNE

Increases in basal ATP release observed in ipsilateral L4/L5 DRG after SNE suggested possible changes in metabolism. We reasoned that increased ATP levels could arise from the demonstrated increases in excitability of both injured and spared sensory neurons [1], which in turn would increase the metabolic demand for ATP needed for the maintenance of ionic gradients. Increased neuronal excitability has been previously linked to increased metabolic activity in spinal cord neurons of normal rats [37], as well as after sciatic nerve ligation [38,39]. However, metabolic changes within sensory ganglia were not examined.

**Figure 5:**
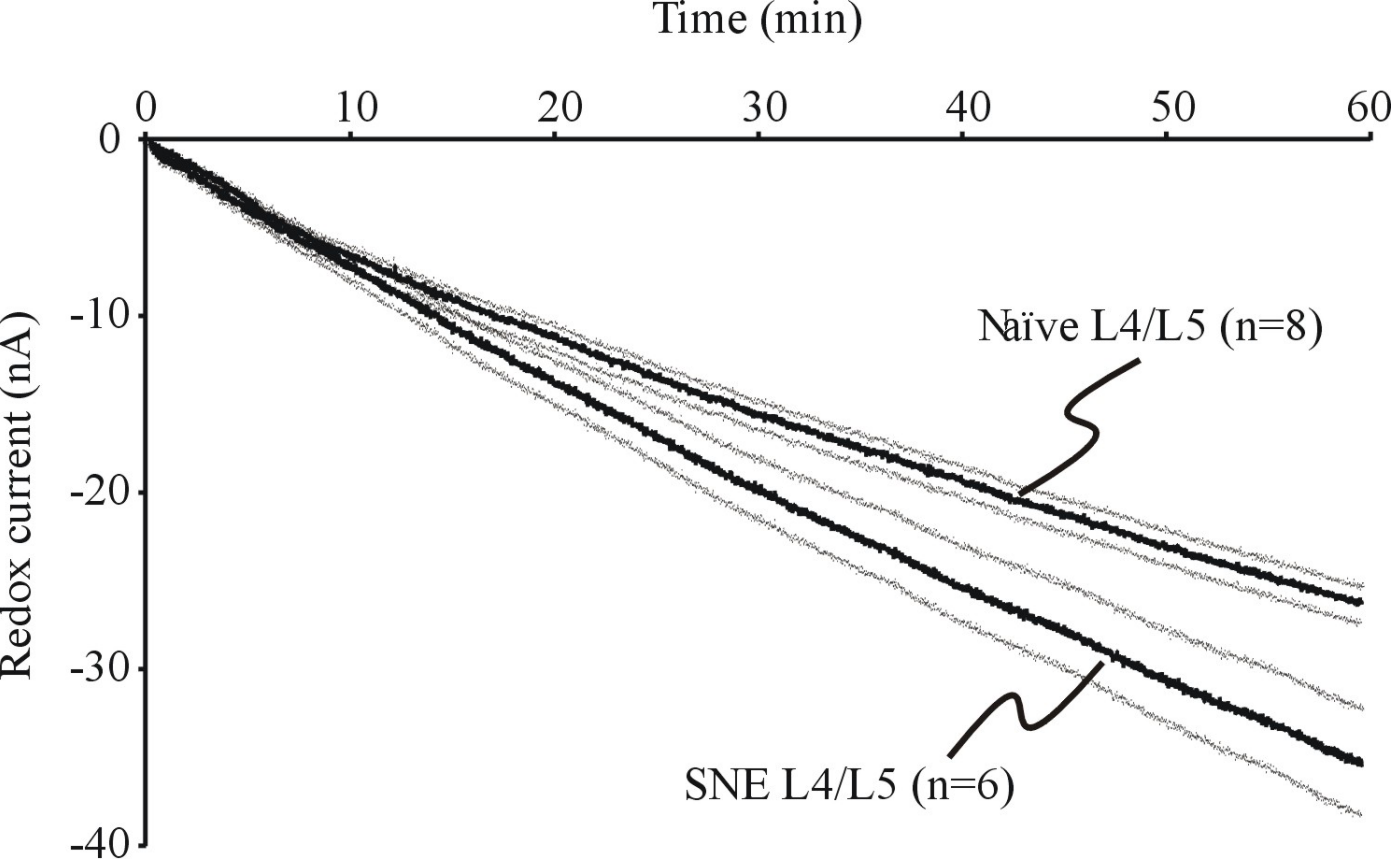
**Increased oxygen consumption of neuropathic DRG**. A: the O_2 _reduction is described as the mean (thick lines) ± SEM (thin lines) redox current (nA) from naive (n = 8) and SNE (n = 6) ganglia. All data were adjusted to 0 nA at time point zero. The gradient of O_2 _reduction (consumption) was significantly faster in DRG ipsilateral to SNE compared to naive DRG (p < 0.05).

To test whether oxidative metabolism is increased in DRG ipsilateral to injury, we adapted the *in vitro *DRG preparation for measurements of O_2 _consumption. Since O_2 _consumption is greatly influenced by factors such as temperature, etc., particular care was taken to ensure similarity of conditions for testing ganglia from different rats. After the ganglia were placed in the testing chamber, the one-hour recording began when temperature increased to a stable level of ≥ 34.5°C. The average temperatures during O_2 _consumption measurements of 34.66 ± 0.15°C (naive DRG) and 34.53 ± 0.20°C (DRG ipsilateral SNE) were not significantly different. DRG ipsilateral to SNE consumed O_2 _faster than control rat DRG (Fig. 5A). The rate of O_2 _consumption (expressed as the rate of negative redox current increase produced by the O_2 _probe) in DRG from naive rats (-24.42 ± 1.14 nA/hr) was significantly different (*p *= 0.005) from that of DRG ipsilateral to SNE (-33.18 ± 2.58 nA/hr) (Fig. 5A). In a separate set of experiments, no significant differences were found in the O_2 _consumption of DRG contralateral to SNE and naive rat DRG (data not shown).

## Discussion

We measured nanomolar ATP levels with the luciferin-luciferase assay after bulk equilibration of perfusate samples with the DRG preparation, permitting relative comparisons of changes in basal and evoked ATP release after neuropathy induction. However, these measurements greatly underestimate the true ATP concentration in the pericellular environment of DRG either during stimulated or basal release, because of its rapid degradation by ectonucleotidases and delayed diffusion due to unstirred layer effects. Recent studies have suggested that true levels of ATP at the plasma membrane are underestimated by >20-fold with bulk phase measurements [9]. Thus, based on measured basal ATP levels in control DRG (Fig. 2A) we would expect true ATP concentration at the neuronal plasma membrane to be >10 nM. At this extracellular [ATP], some degree of P2X3 receptor desensitization would be expected [40,41]. This might explain the paucity of ATP (1–10 mM) responsiveness via P2X3Rs in neurons within intact DRG preparations compared to the robust responsiveness in acutely dissociated neurons [42].

Several possible explanations could account for the increased basal ATP release from DRG ipsilateral to SNE. Previous studies have shown that peripheral denervation (axotomy) leads to progressive degeneration of ~35% of DRG neurons [43,44]. Thus, it is conceivable that breakdown of the plasma membrane of degenerating neurons could lead to spillover of intracellular ATP into the extracellular space where it would contribute to the measured increases in ATP levels. However, after sciatic nerve constriction injury both myelinated and unmyelinated axons are intact proximal to the constriction site despite damage to distal axons, indicating the survival of the DRG neurons whose axons are interrupted [45]. Another possibility which remains to be examined is a neuropathy-induced decrease in the ectonucleotidase-mediated degradation of extracellular ATP [7]. A more plausible explanation might be that higher extracellular ATP levels result from increased excitability of both injured and uninjured neurons [1]. Increased neuronal excitability is clearly linked with increased metabolism [46], which leads to increases in intracellular ATP levels. In yeast [47], as well as mammalian pancreatic β-cells [48-50] and adrenal chromaffin cells [51], increased metabolism and intracellular ATP have been linked with increased extracellular ATP release. Surprisingly, this relationship has not been addressed in central or peripheral neurons. However, we suspect that the hyperexcitability-induced increases in ATP production might result in increased vesicular [52] and non-vesicular release [5] of ATP from somata of DRG neurons.

Evidence in support of this explanation was obtained by demonstrating that O_2 _consumption was increased in L4/L5 ganglia ipsilateral to SNE compared to ganglia from control rats. Almost all of ATP is produced by mitochondria through aerobic respiration. Since O_2 _is not stored to any significant degree by tissues, O_2 _consumption is proportional to energy generation/metabolism. To our knowledge, this is the first time this method was used to examine metabolic changes in sensory ganglia. Previously, the [^14^C]-2-deoxyglucose (2-DG) method was used to study effects of peripheral stimulation in sensory ganglia, but only in normal rats [37]. In these studies, electrical stimulation produced a frequency-dependent increase of glucose utilization in the dorsal horn of the spinal cord, but not within the ipsilateral DRG, leading authors to suggest that axon terminals and not the cell bodies of sensory neurons are the sites of enhanced metabolic activity during increased functional activity of this pathway [37]. Later, spinal cord metabolism was investigated using the 2-DG method in the rat monoarthritis [53] and sciatic nerve ligation [38,39] models. Interestingly, these studies demonstrated increased metabolic activity bilaterally in the spinal cord of arthritic and neuropathic rats, but apparently sensory ganglia were not examined [38,39,53].

Our studies do not differentiate between contributions of the various cell types to extracellular ATP release and metabolism of the entire DRG preparation. Thus, sympathetic fiber sprouting demonstrated by various groups to occur in the DRG in response to sciatic nerve injury, especially after axotomy and deafferentation, [54], could contribute to increased ATP levels via release from sympathetic nerve terminals. Furthermore, while neurons are expected to make a large contribution to extracellular ATP release and O_2 _metabolism, the involvement of other cell types is also likely. Satellite 'glial' cells which form a sheath around the somata of neurons in sensory ganglia undergo proliferation after axotomy or even mild skin abrasions [55]. It is therefore possible that proliferation of satellite cells after SNE contributes to the measured increases in extracellular ATP levels and the increases in O_2 _consumption.

One method used to study neuronal metabolism is the histochemical detection of cytochrome oxidase activity, which is tightly coupled to ATP production [56]. Using this method, chronic decreases in cytochrome oxidase activity were observed after axotomy of neurons within sensory ganglia [57,58]. Loss of peripheral trophic factors and decreased depolarizing activity (action potential discharge) were proposed to contribute to decreased cytochrome oxidase activity in these cells [57]. These studies contrast with our data which indicate increased metabolism and basal ATP release in L4/L5 DRGs after SNE. They also contrast with the demonstrated hyperexcitability of neurons within axotomized DRG in electrophysiological studies [1,59-61]. One likely explanation for the differences is that axotomy results in the loss of a vast majority of the sensory neuron total cytoplasmic mass [62]. Those neurons which survive axotomy have a greatly decreased metabolic demand in maintaining a much shorter peripheral axon.

In addition to increased basal ATP levels, we no longer observed a KCl-evoked release of ATP after SNE. Previously, we used in vivo microdialysis in trigeminal ganglia to show that large increases in extracellular ATP levels result in the apparent loss of KCl-evoked ATP release [6]. Others have demonstrated in different tissues that increases in extracellular ATP lead to increased levels of its various metabolites, including adenosine [63-65]. Also, the release of adenosine from primary sensory neurons is increased in the ipsilateral hindpaw after spinal nerve ligation [31]. Based on these data we reasoned that SNE-induced increases in extracellular ATP levels should also result in increased extracellular adenosine levels. KCl-evoked ATP release *in vivo *is dependent on the activation of voltage-gated Ca^2+ ^channels (VGCCs) [6] and VGCCs are coupled to A1Rs in sensory neurons [36]. A1R activation leads to decreased Ca^2+ ^influx through VGCCs and subsequent decreases in evoked transmitter release in these and other peripheral or CNS neurons [36,66]. Therefore, we hypothesized that the differential SNE effects on basal and evoked ATP release could result from the conversion of extracellular ATP to adenosine with subsequent activation of A1Rs on DRG neurons. To test this hypothesis, we first demonstrated that selective neuronal A1R activation decreases both basal and evoked ATP release in DRG from naïve rats (Fig. 3). Then we demonstrated that selective A1R blockade was able to increase basal ATP levels in DRG both ipsilateral and contralateral to SNE, but more importantly relieved the blockade of KCl-evoked ATP release in DRG ipsilateral to SNE (Fig. 4). These data support our hypothesis that blockade of evoked ATP release is caused by increased A1R activation. An alternative that remains to be explored is that increased P2Y1 receptor activation also contributes to the neuropathy-induced blockade of KCl-evoked ATP release.

The findings of increased metabolism and ATP release in sensory ganglia after peripheral nerve injury have several implications for neuropathic pain mechanisms. Increased extracellular ATP may directly activate neighboring sensory afferents through P2XRs [14,15] thereby increasing nociceptive drive to the CNS. ATP release and P2X-mediated signaling also participate in the enhancement of glutamatergic neurotransmission at the central terminals of primary sensory neurons [67,68]. There is also evidence that peripheral increases in extracellular adenosine may contribute to hyperexcitability of human sensory axons via adenosine A2AR activation [69]. This may be tempered by increased activation of 'inhibitory' A1Rs at peripheral, spinal and supraspinal locations [28,70,71]. Increased activation of P2Y1Rs may also contribute to inhibition of neuronal hyperexcitability [24]. ATP may also potentiate TRPV1 activity, increase axonal transport and increase excitability via activation of P2Y2Rs which likely contributes to nociceptive signaling and hyperalgesia after peripheral nerve injury [72-75]. Thus, SNE neuropathy-induced increases in ATP metabolism and release may contribute to enhanced generation and transmission of nociceptive signals at peripheral terminals, along the axons, within the DRG and at central terminals of primary sensory neurons.

In conclusion, we demonstrated that DRG neurons exhibit increased metabolic rates, increased extracellular ATP levels and increased A1R activation on the side of neuropathy induced by sciatic nerve entrapment. These data add to the literature supporting the use of selective purinoceptor ligands and purine metabolism inhibitors for the therapy of neuropathic pain symptoms [28,76,77].

## Competing interests

The authors declare that they have no competing interests.

## Authors' contributions

YM and IS conceived of, designed, and coordinated the study. YM, SM, BS and IS prepared SNE animals. TO performed behavioral testing and O_2 _consumption experiments. YM, TO, HI, SM, TC, KSO YYNL and BS performed the extracellular sampling and ATP content analysis. YM, TO and BS performed statistical analyses. YM, TO, HI, SM, BS and IS interpreted the results. IS wrote the manuscript. All authors read and approved the final manuscript.
